# Emotion Recognition Ability in Preschoolers: Outcomes of a Socio-Emotional Intervention

**DOI:** 10.3390/brainsci16030269

**Published:** 2026-02-27

**Authors:** Alessandro De Santis, Giusi Antonia Toto, Guendalina Peconio, Annamaria Petito, Pierpaolo Limone

**Affiliations:** 1 Department of Humanities, University of Foggia, 71121 Foggia, Italyannamaria.petito@unifg.it (A.P.); 2 Department of Human Sciences, Education and Sport, Pegaso University, 80143 Napoli, Italy

**Keywords:** emotion recognition, social–emotional learning, DANVA-2, behavioral difficulties, peer problems, early childhood, school-based intervention

## Abstract

**Background**: Emotion recognition ability (ERA) plays a central role in children’s socio-emotional functioning, supporting early social interactions. This study examined whether ERA shows a pre–post change in a classroom-based training context and explored the association between ERA and socio-emotional adjustment. A secondary aim was to compare ERA between children with and without behavioral difficulties. **Methods:** A quasi-experimental study using a controlled non-randomized pre–post design was conducted. The sample included 159 children attending four public elementary schools. Study 1 compared an experimental and a control group assessed before and after the intervention using the DANVA-2-RV. Study 2 examined associations between ERA and behavioral functioning assessed via teacher reports (SDQ-TV) using correlational and group comparison analyses. **Results**: In Study 1, multivariate analyses revealed a significant main effect of Time, indicating overall variation across assessment points, whereas the Time × Group interaction was not statistically significant. Follow-up analyses were therefore interpreted descriptively. In Study 2, lower ERA was associated with higher socio-emotional difficulties, particularly peer problems. **Conclusions**: Across both studies, ERA varied over time regardless of group condition and was linked to socio-emotional adjustment in early childhood. However, the findings do not support a causal interpretation attributing these changes to the intervention. Future randomized studies are needed to determine whether targeted interventions can effectively modify ERA.

## 1. Introduction

Emotions play a central role in human adaptation and involve cognitive processes, physiological changes, and behavioral responses elicited by affectively relevant stimuli [1]. Emotion recognition ability (ERA) is a core process involving the analysis of nonverbal affective signals and attentional orientation to expressive features such as facial configuration and gaze direction. It corresponds to the stage of emotional stimulus identification, prior to regulation and behavioral response [2].

### 1.1. Emotion Recognition Ability as a Cognitive Capacity Within the SEL Skill Model

ERA is a key determinant of social functioning and psychological adjustment [2]. Children’s facial emotion recognition comprises two components: discrimination accuracy, the correct identification of emotions; and response bias, the tendency to assign emotions in the absence of congruent cues (e.g., interpreting neutral or ambiguous expressions as anger) [2,3]. Recognition, as well as the expression of emotions, are two of the fundamental abilities that contribute to success in social interactions [3].

The ability to recognize emotions in facial expressions begins to develop in children starting from the age of two years; around seven years children become progressively more accurate in recognizing facial expressions and this ability shows a stable increase up to adolescence [3,4]. Studies show how 5-year-old children are able to recognize, label and discriminate the intensity of the expression of happiness in a way similar to adults, whereas emotions such as fear are less easily identified [5].

Evidence suggests that contextual and family factors can influence the increase in this ability. A longitudinal study conducted by Milojevich et al. [6] has shown, on a sample of 2232 twins, that children coming from a high socio-economic status recognize facial emotions better; this improvement was attributed to parental abilities (i.e., support, quality of instruction, intrusiveness and maternal warmth) modulating the development of this capacity. The same applies to the school context, where teachers can favor its enhancement. A study by Denervaud and collaborators [7] examined 57 children aged 8–12 years educated for at least six years in Montessori or traditional schools. Montessori children integrated their social context more in the recognition of emotional expressions, and showed a more positive bias. Children from the traditional school instead tended to interpret ambiguous faces as more threatening (greater attribution of fear).

The results indicate that pedagogical practices modulate the development of emotional recognition competences. Such accuracy in emotion recognition is also associated with the quality of peer relationships [8] and is predictive of attitudes in school age [9]. Nowicki and collaborators [10] further highlight that the ability of children aged 8–10 years to deduce others’ emotions from facial expressions, tone of voice and posture is correlated with the locus of control, self-esteem, peer esteem and school achievement.

In McKown’s Social Emotional Learning (SEL) skill model [11], three components are distinguished: nonverbal awareness, social meaning, and social reasoning. Within this framework, social competence functions as a higher-order factor underlying SEL skill and shows significant associations with both emotion regulation and nonverbal awareness. The model further assumes a directional relationship in which SEL skill precedes social behavior. Accordingly, nonverbal awareness is operationalized as ERA, namely the capacity to identify others’ emotions from expressive cues such as facial expressions, body posture, and tone of voice [11]. This conceptualization has guided the design of several school-based Social and Emotional Learning interventions that explicitly target emotion recognition processes.

One example is the PATHS program examined by Domitrovich et al. [12], involving 246 preschool children (mean age ≈ 51 months) from 20 classrooms (10 intervention, 10 control). The intervention consisted of 30 weekly lessons delivered across the school year. Improvements were observed in emotional knowledge, in emotional vocabulary and in perspective-taking abilities; however, however, emotion recognition ability was conceptualized as part of a broader socio-cognitive construct instead of as an operationalized perceptual ability and was therefore oriented toward understanding and using emotional information, with no focus on modifying the processes involved in discriminating expressive signals.

Despite the contribution of McKown’s model and of SEL-based interventions, such as that proposed by Domitrovich [12], the functional level at which the different components of the system operate remains poorly defined. In particular, it is not clear whether the absence of bidirectionality exclusively concerns the processes of self-regulation or also the subcomponents of the SEL skill; nonverbal awareness, social meaning, and social reasoning are treated as a single construct, without specifying whether they represent hierarchically organized stages of social information processing or mutually influencing components of the same competence. As a consequence, the model does not explicitly isolate ERA at the perceptual level. Furthermore, SEL skill interventions predominantly evaluate global behavioral outcomes, making it difficult to establish whether the observed changes depend on modifications of social interpretation processes or on upstream variations in the encoding of emotional signals.

### 1.2. The Role of Emotion Recognition in Emotion Regulation

Emotion regulation (ER) comprises the processes through which individuals modulate their responses to emotionally relevant stimuli. [13]. ER strategies derive from the emotion regulation (ER) model, defined as the set of strategies through which individuals modulate the intensity, duration, or expression of their emotions across social situations [14]. In this sense regulation refers to attempts to influence emotional states circumscribed in time, linked to a specific situation and characterized by a given valence (positive or negative) [15].

Studies on the ER model analyze the effectiveness and adaptiveness of the adopted strategies, evaluating their impact on the achievement of social goals and on long-term well-being [15,16,17]. The ER model proposed by Gross [18] attributes a central role to attentional processes in the regulatory strategies of behavior. From this perspective, several evidence-based interventions, especially in the school context, aim to modify the psychological and neurobiological mechanisms involved in the management of emotions, intervening not only on pupils but also on parents and teachers [18]. Such interventions include activities on the identification of emotional stimuli (for example expressive faces), for the evaluation of affective states [19], and interventions on beliefs related to the malleability of emotions [20].

A recent meta-analysis on facial expression recognition training by Revilla and colleagues [21] highlights improvements in recognition accuracy in developmental clinical samples. However, the authors themselves underline that many interventions are integrated into broader programs that include ER, behavior management and social skills, making it difficult to attribute possible behavioral benefits specifically to the modification of ERA processes [21]. Consequently, the observed changes may reflect contextual or strategic learning rather than a selective modification of the mechanisms of perceptual analysis of the emotional stimulus. In a randomized trial on 186 infants from low-income families, an attachment-based parenting intervention (10 home sessions) was evaluated [22]. The intervention improved caregiver-oriented emotional regulation strategies and reduced emotional dysregulation in the more reactive children.

However, ERA was neither evaluated nor operationalized, since it was an intervention focused on behavioral co-regulation and not on the perceptual discrimination of emotional signals [22]. Despite the theoretical and applicative contribution of the literature on ER, the available models focus predominantly on the processes of the modulation of the affective response once the emotional stimulus has been identified. The stage involving the detection and discrimination of emotional signals, which precedes regulation, remains poorly formalized. As a result, the role of perceptual accuracy in regulatory processes is left implicit, and the functional relationship between emotion recognition and regulation strategies remains unspecified.

### 1.3. Neural Bases of Emotion Recognition

ERA relies on the integration of perceptual, affective, and inferential processing across modalities to support adaptive social behavior [22]. Signals coming from the face, prosody and posture are initially processed in temporo-occipital circuits specialized in the coding of expressive characteristics; subsequently, the amygdala and insula contribute to salience evaluation and to the interoceptive representation of the observed emotional state [22]. Such information then converges in the ventromedial and orbitofrontal prefrontal cortex, where they assume social meaning and guide the selection of the behavioral response [23]. Clinical evidence shows that ventromedial lesions produce alterations of personality and social judgment despite a partial preservation of the perceptual discrimination of expressions [24].

In patients with a traumatic brain injury, deficits of social cognition and emotional recognition are associated with everyday relational functioning even in the absence of general cognitive impairments [25]. Longitudinal studies further indicate that during rehabilitation, recovery proceeds from basic emotions to more complex mental representations, suggesting a progressive functional organization of the socio-emotional network [26]. In subjects of developmental age, early alterations of this system interfere with the acquisition of social competences, highlighting the role of emotional recognition in adaptive development [27].

According to emotion regulation models, prefrontal activity supports the cognitive modulation of affective responses by integrating perceived emotional states with contextual behavioral goals [28]. However, this modulation is not implemented by a single regulatory mechanism: different strategies (detachment, reinterpretation, distraction, and expressive suppression) recruit partially overlapping yet functionally differentiated networks linking prefrontal regions and limbic systems [29].

These regulatory operations presuppose the prior identification of the emotional signal to be regulated. Consistently, electrophysiological evidence shows that specific spatiotemporal EEG patterns allow for the classification of emotional states through frequency-band activity and cortical functional connectivity. Such rapid and distributed neural dynamics index early affective recognition processes that precede full cognitive evaluation and are subsequently integrated by frontal systems involved in social interpretation and regulation [29,30]. In the work of Van Overwalle [31], recognition of emotional states is described as the result of the interaction among three neural systems: the action observation network, mentalizing network and basic limbic network. The posterior cerebellum (Crus I–II and lobule IX) acts as an integration node, exchanging signals reciprocally with the medial prefrontal cortex, pSTS and amygdala. Connectivity analyses show that such circuits support rapid inferences about observed emotional states and the prediction of social sequences [31].

The model therefore suggests a hierarchical processing that starts from the perceptual analysis of expressions and culminates in social understanding, indicating a specific neural role for the discrimination of emotional signals before cognitive interpretation. On the other hand, in a study by Moodie and colleagues [32] based on the ER model, attention is instead directed to the mechanisms of modulation of the affective response. Regulatory strategies (reappraisal, attentional deployment, distancing) mainly activate prefrontal and fronto-parietal regions of cognitive control. Viewing negative stimuli further involves the amygdala, insula, thalamus and associative visual cortices, which constitute the substrate of emotional reactivity on which regulation intervenes. The results therefore indicate a top-down system of modulation subsequent to the evaluation of the stimulus, rather than a mechanism devoted to its perceptual identification [32]. A recent neurophysiological study shows that the processing of emotional expressions is associated with specific electrophysiological markers, in particular the N170 component linked to the structural coding of the face and the LPP associated with the affective evaluation of the stimulus. Kirasirova showed in a sample of 48 adults that these responses are modulated by perceptual experience and attentional allocation, indicating plasticity in emotion recognition mechanisms [33].

However, such paradigms are primarily observational and do not involve structured educational interventions. Conversely, training programs targeting emotion recognition often report behavioral improvements that may reflect task-specific practice effects rather than genuine changes in the underlying ability. Thus, current research separates neural plasticity evidence from intervention-based evidence, leaving unclear whether educational training modifies the neurocognitive mechanisms supporting emotion recognition. This results in a methodological separation between evidence on the neural modifiability of the perceptual process and evidence of the effectiveness of interventions, leaving unexplored the effect of targeted training on emotion recognition ability at the neurocognitive level in typical development.

### 1.4. The Collaborative for Academic, Social, and Emotional Learning Model: Social Awareness and Distinction from Emotion Recognition

The Collaborative for Academic, Social, and Emotional Learning (CASEL) model proposes interventions aimed at the global development of socio-emotional competences in childhood [34,35]. In continuity with the theoretical framework of SEL skill and with emotional regulation models, the CASEL framework defines the general abilities of understanding and management of social and emotional information [36]. Among the five proposed components (self-awareness, self-management, social awareness, relationship skills, and responsible decision-making), social awareness includes the capacity to interpret intentions (theory of mind), the pragmatic meaning of language and others’ emotional states [36]. In this perspective, emotion recognition contributes to the understanding of social interactions and is associated with the quality of interpersonal relationships [36].

However, much of the literature that identifies, in social awareness, the fundamental role of the ability to recognize emotions uses global behavioral indicators, often distinct from specific tasks of perception and the decoding of emotional expressions [37]. In the randomized trial of the Second Step program proposed by Cook and collaborators [38], conducted on 310 teachers and 7419 students, improvements in socio-emotional competences and in school behavior were observed. However, despite the size of the sample, the outcomes were evaluated exclusively through behavioral indicators and teacher-based questionnaires. The absence of direct measures of accuracy in recognizing emotional expressions does not allow us to establish whether the observed changes reflect a modification of social interpretation processes or a variation in the underlying perceptual mechanisms. It follows that the intervention documents effects on global socio-emotional functioning without clarifying the specific contribution of emotion recognition ability. Consequently, emotion recognition is generally treated as a manifestation of overall socio-emotional competence rather than as a specific perceptual–cognitive process. The CASEL framework in fact operates at the level of integrated abilities, without directly operationalizing the mechanisms of analysis of expressive signals, making it uncertain whether school interventions also produce changes on specific cognitive components such as ERA.

Importantly, the existing intervention literature rarely isolates ERA as a distinct outcome, making it difficult to determine whether observed gains reflect genuine changes in recognition ability. Empirical evidence nevertheless indicates that accuracy in recognizing expressions represents a component associated but not overlapping with general socio-emotional functioning [39,40,41,42]. Such ability has been examined in relation to targeted training, but the existing evidence does not disentangle intervention effects from task-specific practice or repeated-exposure effects [43]. Prior studies have reported changes in related performance following interventions based on attention to nonverbal signals and on guided labeling, without however specifically evaluating ERA [44]. From this perspective, since the recognition of expressions depends on processes of perceptual discrimination and emotional categorization, the present study implements structured socio-emotional training aimed at intervening specifically on such ability, considering it distinct, although correlated, with respect to emotional regulation and broader socio-emotional competences [45,46,47].

### 1.5. The Present Study

To fill this gap, the present work considers emotion recognition ability as a perceptual–cognitive process distinct, although correlated, with respect to emotional regulation and broader socio-emotional competences.

The first objective is to examine whether pre–post changes in ERA differ by condition in a classroom-based quasi-experimental design, while considering developmental and repeated-testing effects as plausible influences (RQ1). It was hypothesized that the experimental group (G1) and the control group (G2) would differ in their pre–post change in ERA (H1), with the expected pattern favoring G1.

The second objective concerns the association between the recognition of nonverbal emotional signals and socio-emotional adaptation in the school context (RQ2). It is hypothesized that lower levels of ERA are associated with greater difficulties of socio-emotional adaptation in the classroom context (H2).

The work is articulated in two complementary studies: the first adopts a quasi-experimental pre–post design with a non-randomized control group to evaluate variations in the recognition of emotion in faces; the second uses a cross-sectional design to analyze the association between ERA and socio-emotional adaptation in the school context.

## 2. Materials and Methods

### 2.1. Study Design and Project Description

The Inclusion 4 Children (I4C) project was developed as a collaborative initiative with the Learning Science Institute of the University of Foggia. The study focused on the assessment of emotional competencies in early primary school children, examining ERA in relation to peer interactions and behavioral indicators, including aggressive and bullying-related behaviors. Participation was offered to public inclusive primary schools within the local province through a formal invitation process, and school involvement was voluntary. Participating schools were assigned to either the intervention condition or the comparison condition in a non-randomized controlled design.

Prior to data collection, trainee psychologists involved in the project completed specific training on both the assessment procedures and the intervention protocol to ensure procedural consistency. The present study included only typically developing children.

A non-randomized controlled pre–post design was employed. At baseline, children completed a set of standardized measures. Emotion recognition ability (ERA) was assessed using the Diagnostic Analysis of Nonverbal Accuracy-2 (DANVA-2) (Emory University, Atlanta, GA, USA) and SDQ-TV (SDQ; King’s College London, London, UK)) [39], administered in a revised and standardized version (RV) developed for the present study [47]. Socio-emotional adjustment in the school context was measured using the teacher-reported Strengths and Difficulties Questionnaire (SDQ-TV) [48].

Children assigned to the training condition subsequently took part in a classroom-based training program designed to strengthen emotion recognition skills and to address emotional attributional biases. Children assigned to the control condition remained in regular classroom activities, while teachers participated in brief training meetings on general educational and emotion regulation support strategies, without any procedures specifically targeting emotion recognition. Study outcomes were examined post-test, with analyses primarily focused on changes in emotion recognition ability. The final sample comprised 159 children (82 boys and 77 girls; mean age = 6.0 years, SD = 1.3) and eight female teachers (mean age = 35.0 years, SD = 2.3), recruited from eight first-grade classrooms across four public inclusive schools.

In Study 1, children were assessed pre-test (T0); they completed the classroom-based intervention phase (T1), and were reassessed six months after the end of the intervention (T2). Study 2 adopted a cross-sectional design and included baseline (T0) measures only. Socio-emotional functioning was assessed using teacher-reported questionnaires (SDQ-TV), completed by the classroom teachers.

An a priori power analysis conducted using G*Power version 3.1.9. for a paired-samples t-test (two-tailed), assuming a medium effect size (dz = 0.50), α = 0.05, and power (1 − β) = 0.80, indicated a required sample size of 34 participants. Because the primary Study 1 analysis used a mixed Time × Group design, this power estimate should be considered approximate. A sensitivity analysis with the final sample size (N = 159) showed a minimum detectable effect size of dz = 0.22, indicating adequate sensitivity to detecting small effects.

### 2.2. Procedure

Data collection was conducted by a team of trained graduate psychology students (n = 10) and PhD candidates (n = 3), all of whom had completed prior instruction in standardized child assessment procedures. This training was intended to ensure consistency in test administration and to enhance the reliability of the collected data.

ERA was assessed individually using the DANVA-2-RV, which was administered to each child in a quiet room within the school setting to limit environmental interference and reduce performance-related discomfort. Each assessment session had a duration of approximately 20 to 25 min. To prevent fatigue, testing was distributed across multiple days, with children participating in a single session per day. At the start of each session, the study was introduced to the children in age-appropriate language, emphasizing both the confidentiality of the procedures and the voluntary nature of their participation.

Following the completion of the individual assessment, teachers were provided with a sealed envelope containing a QR code granting access to the online version of the SDQ-TV. Teachers completed the SDQ-TV independently at a later time, outside the testing context. This sequencing was adopted to ensure that teacher ratings were not influenced by children’s test performances and to allow completion under low-pressure conditions. Written informed consent was obtained from the legal guardians of all participating children. Participation was voluntary, and all data were anonymized and processed in accordance with institutional data protection regulations.

#### 2.2.1. Rationale of the Classroom-Based Training

The training was implemented in accordance with the Universal Support level (Tier 1) of the CASEL framework [35], therefore being addressed to the entire class. Unlike SEL programs, the activities were designed to intervene selectively on the processes of the analysis of emotional signals. In particular, the operative components followed a functional progression: (a) emotional categorization, aimed at the stabilization of perceptual categories; (b) recognition of nonverbal facial signals, aimed at the coding of expressive characteristics; (c) discrimination of intensity, aimed at the fine calibration of perceptual sensitivity; and (d) guided reflection on the stimulus–emotion–behavior association, aimed at the use of the recognized information. Each component was operationalized through structured classroom activities, constantly maintaining the objective of intervening primarily on recognition accuracy rather than on the teaching of regulation strategies or general social competences.

#### 2.2.2. Implementation of the Classroom-Based Training in the Groups

The classroom-based training was carried out in the classroom context and structured into five sessions of approximately 90 min each, conducted in the presence of the main teachers with a managerial support function. The sessions took place on alternate days during school hours and were administered to all classes in the same order. The activities were designed to intervene progressively on different levels of the processing of emotional signals: categorization, recognition, discrimination of intensity and use of emotional information.

Session 1. Introduction and emotional categorization. The first session introduced the concept of emotion and the basic emotions. Through a role-playing activity [49], three children simulated a frustrating social situation. Participants were required to identify the emotion experienced and propose appropriate behavioral modalities. A guided discussion followed in which the strategies that emerged were classified as functional or dysfunctional through an interactive whiteboard.

Session 2. Recognition of nonverbal facial signals. Using cards depicting seven basic emotions (happiness, sadness, anger, fear, worry, disgust, surprise) [49], one child reproduced the facial expression while peers identified it verbally. After identification, a personal example associated with the emotion was requested. The activity was aimed at coding the expressive signals of the face.

Session 3. Discrimination of emotional intensity. On an interactive whiteboard, emotional emojis with different intensities were presented. Children attributed to each a level (low, medium, high) through graphic thermometers. Twenty trials were proposed (5 per emotion) with immediate corrective feedback.

Session 4. Discrimination of intensity with focus on fear. Children evaluated the emotional intensity evoked by neutral images and images of potentially dangerous animals using graded categories (“not at all”–“very much”). Subsequently they described the possible behavioral reaction; the operator clarified the association between stimulus, emotion and response.

Session 5. Stimulus–emotion–behavior association. Through five short stories three standardized questions were posed: emotion of the protagonist, adequacy of the behavior and the child’s personal response. The answers were discussed collectively with explicit feedback.

The control group carried out the function of an active observational cohort. The teachers participated in five training meetings (1 h each) on secondary and tertiary educational strategies proposed by Harrington [15]. The training concerned support to peer interactions, guided emotional discussion and regulation strategies, without structured activities directed to the children nor specific exercises on the recognition of emotional expressions. The children therefore did not receive any training on the target ability.

### 2.3. Sample

#### 2.3.1. Sample Study 1

As shown in Table 1, the sample consisted of 159 children attending four inclusive public elementary schools. The mean age of the participants was 6 years (SD = 1.3). The sample included 82 males (51.6%) and 77 females (48.4%), indicating a balanced sex distribution. Participants were assigned by classroom to an experimental group (n = 81 at pre-test; n = 77 at post-test) and a control group (n = 78), resulting in comparable group sizes.

Children were drawn from eight classes: Class 1 (n = 20), Class 2 (n = 9), Class 3 (n = 11), Class 4 (n = 22), Class 5 (n = 19), Class 6 (n = 28), Class 7 (n = 26), and Class 8 (n = 24). For analytical purposes, two levels of analysis were defined. The full sample (N = 159) was retained for analyses not requiring distributional assumptions.

For parametric analyses, a parametric subsample was derived by retaining cases meeting distributional criteria and excluding statistical outliers, resulting in n = 50 in the experimental group and n = 59 in the control group. This procedure did not alter study participation but determined which observations entered specific statistical models. The study procedure was fully completed in three classes (Classes 1–3); these 40 children (25.2% of the total sample) constituted the sample for Study 2.

#### 2.3.2. Sample Study 2

The analytic sample, summarized in Table 2, included 40 first-grade children drawn from three classrooms. Children had a mean age of 6.0 years (SD = 0.44) and comprised 21 boys and 19 girls. Three female teachers participated in the study (mean age = 35.0 years, SD = 2.3), each with substantial experience in early education (mean years of service = 12, SD = 4.4). The sample was characterized by cultural diversity, with 28 children of Italian origin and 12 children from other ethnic backgrounds, including North African (n = 4), Romanian (n = 6), and Turkish (n = 2) origins. With respect to parental education, the majority of families reported low educational attainment (78%), followed by medium (20%) and high (2%) levels. Based on established cut-off criteria of the Strengths and Difficulties Questionnaire–Teacher Version, children were categorized into a non-clinical group (n = 17) and a clinical group (n = 23).

### 2.4. Assessment

#### 2.4.1. DANVA-2-RV

**Figure 1:**
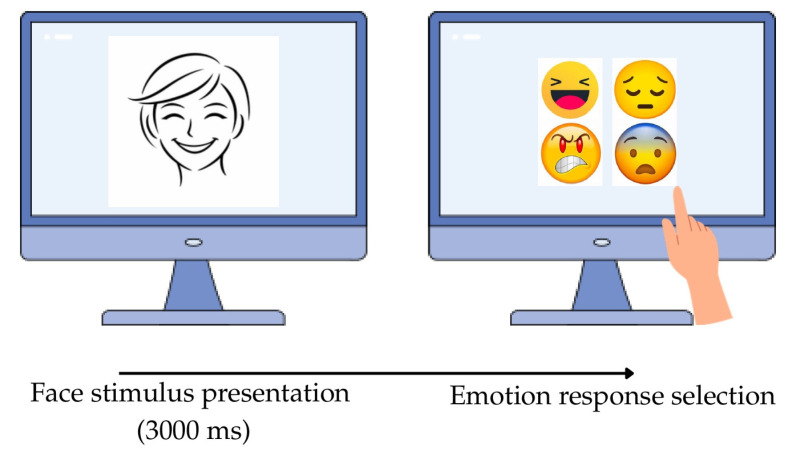
Experimental task procedure. In each trial, an emotional face or posture stimulus was presented for 3000 ms, followed by a response screen with emoji icons and written emotion labels. Participants selected the emotion that best matched the perceived expression.

The DANVA-2-RV was administered in a research adaptation for classroom use. Responses were collected through a touchscreen pointing procedure with four pictorial response icons representing happiness, sadness, anger, and fear. The task was administered individually on a touchscreen laptop. Children sat in front of the device and viewed an emotional stimulus presented for 3000 ms, followed by a response screen displaying the four alternatives. The spatial position of the response icons was randomized across trials. Participants selected the perceived emotion by directly touching the corresponding icon (see Figure 1).

Visual presentation parameters were adapted to a digital format to facilitate classroom administration. The stimulus set included child faces, adult faces, and body postures (24 child faces, 24 adult faces, 12 postures), presented at two intensity levels across the four basic emotions (15 items per emotion). Scoring followed the standard DANVA-2 accuracy procedure, yielding an overall emotion recognition score computed as the sum of correct responses. Psychometric properties and a validation of the original instrument are reported in the test manual [39] and related publications [47]; in the present study, the posture stimuli were updated with more recent images while maintaining the original emotional categories and scoring procedures of the standardized instrument.

#### 2.4.2. SDQ-TV

Children’s socio-emotional behavior was evaluated using the SDQ-TV, a standardized measure of behavioral difficulties and prosocial attitude in school contexts. The questionnaire includes five subscales assessing emotional symptoms, conduct problems, hyperactivity, peer problems, and prosocial attitude. Teachers rated each item on a three-point scale, and a total difficulties score was computed by summing the four problem-related subscales. The SDQ-TV demonstrates good internal consistency for the total score (α = 0.90) and acceptable-to-good reliability across subscales [48]. In addition to dimensional scores, established cut-offs were used to classify children’s level of socio-emotional difficulties [48].

### 2.5. Data Analysis and Data Administration

Data were screened and analyzed using MATLAB (R2024b The MathWorks, Inc., Natick, MA, USA). Prior to the main analyses, datasets were inspected for completeness and distributional properties (see Code in Supplementary Materials for details). Outliers were identified using the boxplot interquartile range criterion and removed only for analyses requiring parametric assumptions. Normality was evaluated using the Shapiro–Wilk test. When violations emerged, a Box–Cox transformation was applied; in variables containing zero values that prevented transformation, zero scores were removed. If normality could not be achieved after these procedures, cases were excluded from the parametric subsample but retained in the full dataset for distribution-free analyses.

All participants were retained in the study; filtering procedures affected only the eligibility for parametric analyses. The total sample consisted of eight classes (N = 159). Classes 1–5 formed the experimental group (n = 81) and Classes 6–8 the control group (n = 78). The full dataset (N = 159) was used for non-parametric analyses, whereas a parametric subsample was derived exclusively for models requiring distributional assumptions. The parametric experimental subsample included Classes 1–4 (n = 62), from which 12 outliers were excluded (final n = 50). The parametric control subsample included Classes 6–8 (n = 78), from which 19 outliers were excluded (final n = 59) following the same distributional screening criteria applied to the experimental group, ensuring comparability under parametric assumptions. Exclusion did not modify group allocation or participation status, but only the observations entering parametric models.

For Study 1, analyses followed a hierarchical strategy. First, a multivariate repeated-measures analysis of variance (MANOVA) was performed on the parametric subsample with Time (pre, post) as a within-subject factor and Group (experimental, control) as a between-subject factor, using the DANVA-2-RV subscale scores as dependent variables, in order to test the overall temporal effect and the Time × Group interaction. A principal component analysis was conducted to evaluate whether the emotion recognition measures supported the use of a global total score. Pre–post changes were then described using paired-sample t-tests and non-parametric tests (Wilcoxon signed-rank tests; Mann–Whitney U tests on change scores), with group differences additionally described post-assessment. These analyses were conducted on the full dataset and interpreted as descriptive follow-ups.

For Study 2, analyses focused on the subsample for which teacher-reported data were available. Socio-emotional adjustment was assessed using the SDQ-TV, and participants were classified into clinical and non-clinical groups based on established cut-off scores. Group comparisons were conducted to examine differences in emotional recognition performance between children with and without socio-emotional adjustment difficulties.

Statistical significance was set at α < 0.05 (two-tailed) for all analyses.

## 3. Results

### 3.1. Assumption Checking for Study 1

Prior to conducting the main analyses, the assumptions underlying the applied statistical procedures were systematically examined. These included checks of univariate and multivariate normality, linearity, homogeneity of variance and variance–covariance matrices, absence of multicollinearity, and identification of potential outliers. All assumptions were adequately met for the analyses reported in the main text. Detailed results of assumption checks are provided in the Supplementary Materials. No adverse effects or unintended effects were observed in either study condition.

#### Results of Study 1

**Figure 2:**
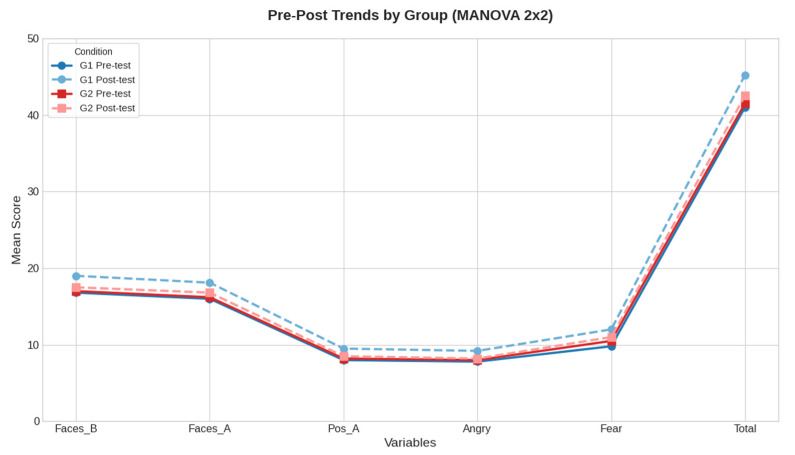
Mean pre–post scores across DANVA-2-RV subscales for experimental (G1) and control (G2) groups. Solid lines represent pre-test scores, whereas dashed lines represent post-test scores.

The multivariate repeated-measures analysis revealed a significant main effect of Time (Pillai’s Trace = 0.048, F(1, 98) = 4.91, *p* = 0.029), indicating an overall change over time across both groups. The Time × Group interaction was not significant (*p* = 0.053). The pattern of means across conditions is shown in Figure 2.

**Figure 3:**
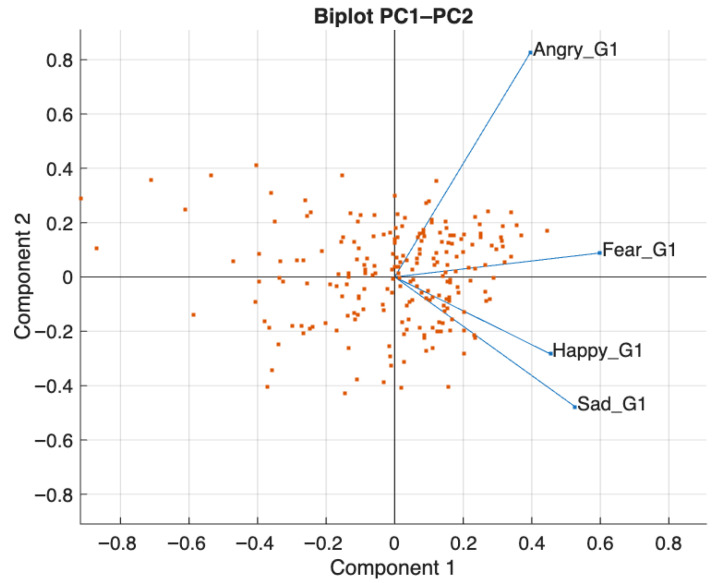
PCA of DANVA-2-RV subscales showing a single dominant component supporting the use of the total score.

A principal component analysis (PCA; Figure 3) was conducted on the emotion recognition variables to examine their component structure. A single dominant component accounted for approximately 80% of the variance, with all variables loading positively (fear = 0.60, sadness = 0.53, happiness = 0.45, anger = 0.40), indicating that the subscales shared substantial common variance in this dataset and supporting the use of a global total score for subsequent descriptive analyses. Given the non-significant Time × Group interaction, the following analyses are reported as descriptive follow-up analyses.

**Figure 4:**
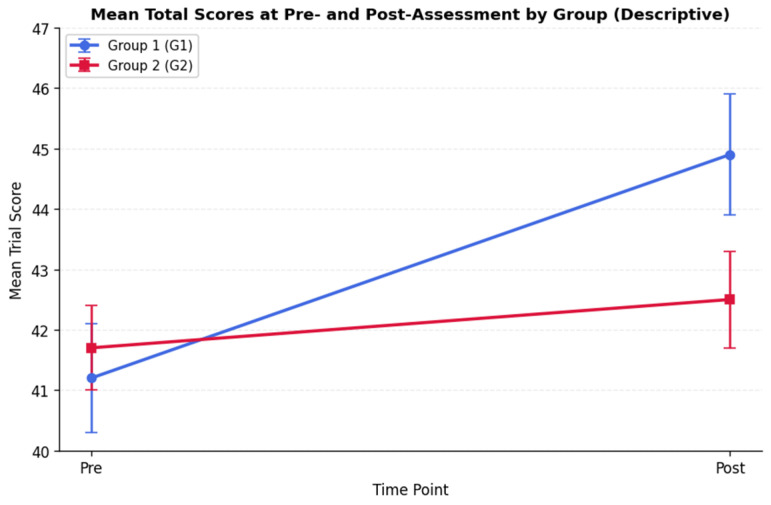
Mean total scores pre- and post-assessment for the experimental group (G1) and control group (G2), shown for descriptive purposes only. Error bars represent 95% confidence intervals. In the primary inferential model, the Time × Group interaction was not statistically significant; therefore, this figure should not be interpreted as evidence of a differential intervention effect.

Descriptively, only Group 1 showed a pre–post increase in the Total score (t = −2.70, *p* = 0.010), whereas Group 2 did not (*p* > 0.05). Post-assessment, groups differed on the Total score (t = 1.87, *p* = 0.045). Non-parametric analyses on the Total score showed the same descriptive pattern as the parametric results. In Group 1, the Wilcoxon signed-rank test indicated a pre–post increase (*p* < 0.001; median Δ = +5), whereas Group 2 showed no change (*p* = 0.626; median Δ = 0). As a descriptive follow-up analysis, we conducted a Mann–Whitney U test on pre–post change scores, which showed a difference in change-score distributions across groups (*p* = 0.0019). Given the non-significant Time × Group interaction in the primary inferential model and the classroom-based non-randomized design, this result is reported descriptively and should not be interpreted as evidence of a differential intervention effect. Figure 4 displays mean Total scores pre- and post-assessment for both groups.

### 3.2. Results of Study 2

Pearson’s r coefficients were computed to examine the association between emotional recognition ability (DANVA Total score) and behavioral outcomes measured by the Strengths and Difficulties Questionnaire (SDQ). As reported in Table 3, a higher emotional recognition performance was associated with lower levels of behavioral and emotional difficulties. Specifically, the DANVA Total score showed a moderate negative correlation with overall SDQ difficulties (r = −0.34, *p* < 0.01) and with the Emotional Problems subscale (r = −0.30, *p* < 0.05). A strong negative association was observed with peer problems (r = −0.57, *p* < 0.001), indicating that poorer emotional recognition ability was related to greater difficulties in peer relationships. Negative associations were also observed with conduct problems (r = −0.24) and hyperactivity (r = −0.14); however, these correlations did not reach statistical significance, although they followed the same directional pattern observed for the other difficulty-related subscales. In contrast, emotional recognition ability was positively associated with prosocial attitude (r = 0.25), suggesting that better emotion recognition is related to higher levels of prosocial functioning. Overall, the correlational pattern suggests that lower emotional recognition ability is associated with greater socio-emotional difficulties, particularly in the domain of peer relationships, while showing a positive relationship with prosocial attitudes.

**Figure 5:**
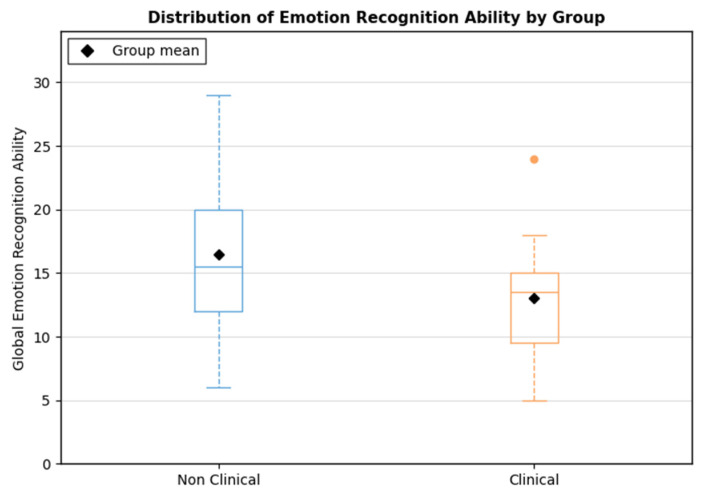
Distribution of global emotion recognition ability by group. Blue boxes represent the Non-Clinical group, and orange boxes represent the Clinical group. The black diamond indicates the group mean.

Prior to analysis, assumptions for multivariate testing were examined and met, including multivariate normality, linearity, homogeneity of variance–covariance matrices, and absence of multicollinearity or multivariate outliers. Participants were classified into clinical and non-clinical groups based on established SDQ total difficulties cut-offs [48]. A one-way ANOVA revealed a significant group difference in total emotion recognition scores (F(1, 38) = 4.65, *p* = 0.037), with children in the clinical group showing lower recognition accuracy than their non-clinical peers (Figure 5). These results suggest that clinically significant behavioral difficulties are associated with poorer emotion recognition performance.

**Figure 6:**
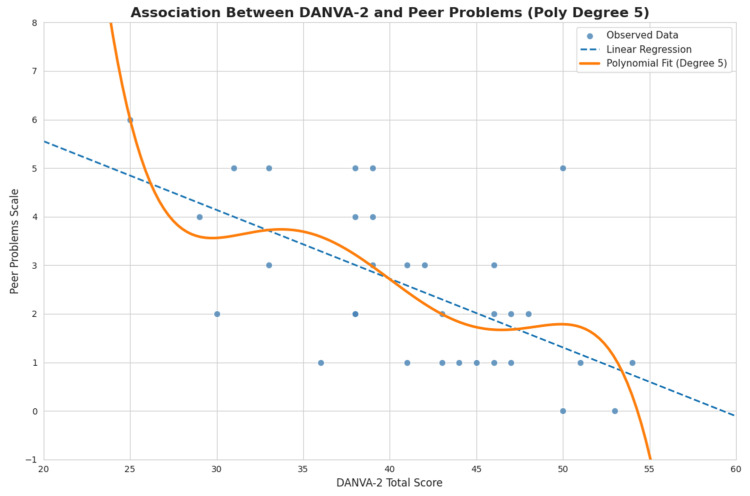
Association between emotional recognition ability and peer problems. Scatterplot illustrating the relationship between DANVA-2 Total score and peer problems scale scores. The solid line represents the linear regression fit, while the dashed line represents the polynomial fit regression model.

To further explore the association between emotional recognition ability and peer-related difficulties, linear and quadratic regression models were tested with the DANVA-2 Total score as predictor and the peer problems scale as outcome (Figure 6). The linear regression model revealed a significant negative association between DANVA-2 Total score and peer problems, β = −0.14, SE = 0.03, *p* < 0.001, indicating that a higher emotional recognition ability was associated with fewer peer-related difficulties. The model accounted for approximately 33% of the variance in peer problems (R^2^ = 0.33). A polynomial term was subsequently included to test for potential non-linear effects. However, the exploratory polynomial (Degree 5) model did not provide a meaningful improvement in model fit compared to the linear solution, suggesting that the relationship between emotional recognition ability and peer problems is adequately described by a linear trend across the observed score range.

## 4. Discussion

The initial hypothesis predicted that the training would produce an improvement in the experimental group compared to the control group. The multivariate repeated-measures analysis showed a significant main effect of Time, indicating an increase in recognition accuracy across groups (*p* = 0.029). However, the Time × Group interaction was not statistically significant. Therefore, the present findings do not provide inferential evidence of a differential intervention effect. Observed pre–post differences may reflect developmental processes, repeated-testing effects, contextual influences, or a combination of these factors. Consequently, group comparisons and follow-up analyses were interpreted descriptively, rather than as confirmatory evidence of a differential intervention effect. In this descriptive context, total scores differed post-test (*p* = 0.045). Overall, the findings are compatible with change over developmental time in emotion recognition ability, while the observed pattern of change cannot be specifically attributed within the present design. This result is consistent with the literature showing that emotion recognition becomes progressively more refined over developmental time [50,51,52]. The training included several specific (e.g., labeling, intensity discrimination) and non-specific (e.g., perspective-taking, progressive exposure) activities. Accordingly, the observed changes are interpreted as time-related changes, and the present design does not allow the contribution of individual intervention components to be isolated.

The second hypothesis concerned the relationship between emotional recognition and social functioning. A strong negative association with peer difficulties emerged (r = −0.57), indicating that lower perceptual accuracy was associated with greater relational problems. This pattern was also reflected in the SDQ-based clinical/non-clinical group comparison. Peer problems showed the strongest association with variability in recognition accuracy in the present sample. The results support an association between the quality of emotion recognition and peer-related functioning, without establishing a directional pathway. Similar associations have been observed both in clinical and non-clinical samples, suggesting that such a relationship may reflect a broader feature of socio-emotional functioning rather than a specific population effect [52].

Considered jointly, the results indicate that emotion recognition does not act as a global social ability but as a perceptual process associated with relational functioning. Study 1 shows that perceptual accuracy changes over time, while the observed pattern cannot be specifically attributed within the present design; Study 2 shows that such accuracy is associated with the quality of peer interactions. Taken together, the findings support an association between socio-emotional functioning and emotion recognition accuracy. Although associations with relational functioning were observed, the present design does not allow intervention-specific effects or underlying mechanisms to be conclusively established.

However, several limitations of the present study should be acknowledged. A first limit of the present work concerns the aggregated nature of the measures used, which does not allow us to describe the temporal dynamics of the processing of emotional signals. Future studies based on process measures (e.g., eye-tracking or neurophysiological EEG signals) could benefit from advanced computational approaches. In such contexts, machine learning methodologies, already successfully applied to the analysis of electrophysiological signals, allow for the modeling of complex and non-linear patterns that are difficult to capture through traditional statistics, for example through data augmentation techniques, semi-supervised learning or the integration of multiple classifiers. Such approaches do not replace the inferential analyses used in the present study, but represent a natural extension when the object of investigation becomes the microtemporal dynamics of perceptual processes [53].

With regard to Study 1, several limitations should be acknowledged. Although the post-assessment was conducted six months after the end of the intervention, the present design does not allow the source of change to be determined, and the observed pre–post differences may reflect developmental change, repeated-testing effects, intervention-related factors, or a combination of these influences. In addition, allocation was non-randomized and classroom-based, and class-level clustering was not modeled analytically, which further limits causal inference. Finally, the intervention was multicomponent and was not exclusively focused on emotion recognition; therefore, the contribution of individual program components to the observed changes in related socio-emotional competences cannot be isolated.

The second study, of a cross-sectional nature, instead does not allow definitive causal inferences regarding the relationship between emotional recognition and relational functioning. Although a sequential interpretation is plausible, it was not tested in the present cross-sectional study. Furthermore, the measures used are predominantly based on behavioral instruments and questionnaires, which reflect observable functioning but do not allow us to precisely distinguish the underlying cognitive processes; therefore the interpreted mechanisms must be considered at a functional rather than strictly neurocognitive level.

Finally, the sample considered, although including different levels of socio-emotional vulnerability, does not necessarily represent the entire variability of the school population and limits the generalizability of the results to other cultural contexts or different age ranges.

## 5. Conclusions

Overall, the two studies address complementary aspects of ERA: its change over time and its functional relevance. Study 1 indicates time-related variation in ERA across assessment points, without evidence of differential change between experimental and control conditions. Study 2 shows that individual differences in perceptual accuracy are associated with the quality of socio-emotional adaptation in school contexts.

While considering the limits of the cross-sectional design and the dropout in the collection of teachers’ evaluations, the results indicate that ERA can be interpreted as a perceptual component with relational implications rather than as a global social competence. In this perspective, future studies should examine the temporal relationship between variations in perceptual accuracy and socio-emotional adaptation, as well as integrating process measures at the microtemporal level in order to clarify how attentional patterns toward expressive signals contribute to the observed individual differences.

## Figures and Tables

**Table 1:** Sample description and analysis subsets.

Index	n	% (N = 159)
Children	159	100.0
Mean age	6 (±1.3)	
Sex		
Male	82	51.57
Female	77	48.42
Sample partitioning		
Experimental group	81	50.94
Control group	78	49.05
Group allocation (by classroom) ^1^		
Class 1	20	12.57
Class 2	9	5.66
Class 3	11	6.92
Class 4	22	13.48
Class 5	19	11.95
Class 6	28	17.61
Class 7	26	16.35
Class 8	24	15.09
Parametric subsample–experimental group ^2^	50	
Parametric subsample–control group ^3^	59	
Full dataset	159	
Classes participating in Study 2		% (N = 159)
Class 1	20	12.57
Class 2	9	5.66
Class 3	11	6.92
Study 2 sample	40	25.15

^1^ Classes 1, 2, 3, 4, 5 were included in experimental group; ^2^ Classes 1, 2, 3, 4 were included in normative experimental sample; 12 outliers were excluded from normative experimental group; ^3^ Classes 6, 7, 8 were included in normative control group; 19 outliers were excluded from normative control group.

**Table 2:** Sample Study 2 description.

Variable	n	% (N = 40)
Total children	40	100.0
Mean age (years)	6 (±0.44)	
Sex		
Male	21	52.5
Female	19	47.5
Ethnic background		
Italian	28	70.0
North African	4	10.0
Romanian	6	15.0
Turkish	2	5.0
Parental education		
Low	31	78.0
Medium	8	20.0
High	1	2.0
Number of classes		
1	20	50.0
2	9	22.5
3	11	27.5
Teachers		
Sex (female)	3	100.0
Mean age (years)	35 (±2.3)	-
Years of service	12 (±4.4)	-
SDQ classification		
Clinical (total score > 15)	23	57.5
Non-clinical (total score < 15)	17	42.5

**Table 3:** Pearson correlations between DANVA-2-RV Total score and SDQ subscales.

Variable Pair	r (*p*-Value)
DANVA Tot. Score ^1^—SDQ Total Difficulties	−0.34 **
DANVA Tot. Score—Emotional Problem	−0.30 *
DANVA Tot. Score—Conduct Problem	−0.24
DANVA Tot. Score—Hyperactivity	−0.14
DANVA Tot. Score—Peer Problem	−0.57 ***
DANVA Tot. Score—Prosocial Attitude	+0.25

^1^ Higher DANVA scores indicate better emotional recognition ability. Negative correlations reflect greater behavioral difficulties associated with lower emotional recognition performance. *p* < 0.05 *, *p* < 0.01 **, *p* < 0.001 ***.

## Data Availability

The original contributions presented in this study are included in the article/Supplementary Materials. Further inquiries can be directed to the corresponding author.

## Supplementary Materials

The following supporting information can be downloaded at: https://www.mdpi.com/article/10.3390/brainsci16030269/s1, Table S1: TREND Reporting Checklist and Participant Flow Diagram; Figure S1: Participant Flow Diagram; Database; Code. Ref. [54] was cited in Supplementary Materials. The supplementary material provided with this article includes the complete anonymized dataset used in the analyses, encompassing both the normative and non-normative samples as well as the SDQ subsample. In addition, the supplementary files contain the MATLAB scripts employed to conduct all statistical analyses, together with detailed outputs reporting descriptive statistics, parametric and non-parametric results, and all procedures used to verify statistical assumptions. These materials are provided to ensure transparency of data processing and analytical methods and to allow independent verification and replication of the findings.
